# Effects of Combined Creatine and Sodium Bicarbonate Supplementation on Soccer-Specific Performance in Elite Soccer Players: A Randomized Controlled Trial

**DOI:** 10.3390/ijerph18136919

**Published:** 2021-06-28

**Authors:** Jooyoung Kim

**Affiliations:** Office of Academic Affairs, Konkuk University, 268, Chungwon-daero, Chungju 27478, Korea; hirase1125@hanmail.net; Tel.: +82-43-840-3520

**Keywords:** agility, creatine, soccer player, sodium bicarbonate, sprint

## Abstract

Creatine and sodium bicarbonate are both ergogenic aids for athletic performance. However, research on the combined creatine and sodium bicarbonate (CSB) supplementation in soccer is limited. This study investigated the changes in soccer-specific performance in elite soccer players after supplementing with CSB. Twenty well-trained elite soccer players participated in the study (age: 20.70 ± 1.08 years; height: 173.95 ± 2.81 cm; body weight: 70.09 ± 3.96 kg; soccer experience: 8 years; average training hours per week: 20 h). The participants were randomly allocated into CSB groups (CSB, *n* = 10) and placebo groups (PLA, *n* = 10). The CSB group took creatine (20 g/day) and sodium bicarbonate (0.3 g/kg/day); these two supplements were taken four times a day (morning, afternoon, evening, and before sleep) for seven days. Soccer-specific performance was assessed via 10- and 30-m sprint, coordination, arrowhead agility, and Yo-Yo intermittent recovery level 1 tests. Compared to the PLA group, the CSB group performed better in the 30-m sprint (CSB: −3.6% vs. PLA: −0.6%, *p* = 0.007, effect size (ES): 2.3) and both right and left arrowhead agility (right: CSB: −7.3% vs. PLA: −0.7%, *p* < 0.001, ES: 2.8; left: CSB: −5.5% vs. PLA: −1.2%, *p* = 0.001, ES: 2.1) tests. However, there were no differences in 10 m sprints, coordination, and Yo-Yo intermittent recovery level 1 tests between the two groups (*p* > 0.05). In conclusion, CSB supplementation improved sprint and agility in elite soccer players. However, it is still unclear whether such effect is synergistic effect of two supplements or the result of either one of them. Therefore, caution should be taken when interpreting the results, and the limitations should be examined further in future studies.

## 1. Introduction

Soccer is a global sport with 203 member nations in the Federation of International Football Association (FIFA) and is one of the most popular sports in the world [1]. During a 90-min game, soccer players run an average distance of 10 km, intermittently approach the anaerobic threshold of ~90% maximal heart rate, and are required to perform many explosive movements [2]. Performance in a soccer game is influenced by various factors, such as technical, tactical, mental, physiological, and biomechanical aptitude [3,4].

Therefore, coaches, trainers, and players are interested in various factors for optimizing performance in soccer games, especially apparel, footwear, training, diets, and ergogenic aids that can help improve and maintain player performance in a game [5,6]. Among them, ergogenic aids are useful tools that can be utilized for improving performance in sports [7]. Through a consensus statement, the International Olympic Committee (IOC) recently presented few ergogenic aids that have adequate level of scientific evidence and are safe and effective with respect to performance gains by athletes. Such aids include caffeine, creatine, nitrate, sodium bicarbonate, and beta-alanine [8].

Creatine is naturally occurring non-protein amino acid found in red meat and seafood [9], which is among the most popular ergogenic aid for athletes [10]. Creatine supplementation increases intramuscular creatine concentrations and phosphocreatine (PCr) stores, improving the capacity of the phosphagen system to supply adenosine triphosphate (ATP) under anaerobic conditions, which has been shown to enhance components of physical performance in soccer players [11,12,13]. Claudino et al. [11] reported that 7 weeks of pre-season creatine supplementation improved soccer players’ jump performance. Mujika et al. [12] reported that 6 days of creatine supplementation improved repeated sprint performance and jumping ability. Yáñez-Silva et al. [13] also reported that 14 days of creatine supplementation increased the muscle power output of young soccer players.

Meanwhile, player performance decreases as blood pH, and bicarbonate levels decrease throughout a game [14]. Thus, supplementing buffering agents, such as sodium bicarbonate (NaHCO_3_), may reduce metabolic acidosis and improve anaerobic performance during exercise [15,16]. Recently, Chycki et al. [15] demonstrated that soccer player’s sprint performance significantly improved over repeated bouts after supplementing sodium bicarbonate for 9 days. Moreover, Krustrup et al. [17] and Wang et al. [18] have found that sodium bicarbonate supplements increase blood pH and bicarbonate concentrations, improving athletic performance [17,18].

As described earlier, creatine and sodium bicarbonate can contribute to improving athletic performance through different mechanisms in intracellular and extracellular environment [11,12,17,18]. Considering that both creatine and sodium bicarbonate could theoretically function as a cellular buffer and have the physiological mechanisms that PCr hydrolysis consumes a H+ ion and buffer acidosis [9,11,15], it is believed that synergistic effect could be expected by combining these two supplements. Recently, Sarshin et al. [9] reported that CSB supplementation enhanced peak power and mean power in Taekwondo athletes. Barber et al. [19] showed that CSB supplementation in trained men improved peak and mean power and attenuated peak power decline. Moreover, Mero et al. [20] reported that CSB supplementation improved consecutive maximal swim performance in competitive male and female swimmers. These results suggest that CSB supplementation can positively affect athletic performance; however, research on soccer is still limited. 

Therefore, this study aimed to investigate the effect of CSB supplementation on soccer-specific performance in elite soccer players. This study hypothesized that CSB supplementation might improve the performance of elite soccer players in 10- and 30-m sprints, coordination tests, arrowhead agility tests, and Yo-Yo intermittent recovery level 1 tests.

## 2. Materials and Methods

### 2.1. Subjects

The study involved 20 elite soccer players (6 defenders, 9 midfielders, 5 forwards) aged 20.70 ± 1.08 years; height 173.95 ± 2.81 cm; and body weight 70.09 ± 3.96 kg. G-power program (ver. 3.1.3, Heinrich-Heine-University, Düsseldorf, Germany) was used to calculate the sample size, with settings of statistical power (1-β) of 0.80, alpha error probability of 0.05 and an effect size of 0.40. The average length of soccer experience was 8 years. The average training hours per week was 20 h. The subjects did not receive any nutritional supplements in the prior 3 months and did not have musculoskeletal injuries. The subjects were randomly assigned to the CSB supplementation (creatine: 20 g/day; sodium bicarbonate: 0.3 g/kg/day) group (CSB, *n* = 10) or placebo group (PLA, *n* = 10), consisting of maltodextrin. A web-based random number generator (http://www.random.org (accessed on 23 November 2020)) was used for assignment to the CSB or placebo group. The physical characteristics of the subjects in each group are shown in Table 1. The study was conducted during the competitive offseason. During the study, all subjects lived in the team dormitory and received the same training and meals. After being informed of the study purpose, experimental procedures, possible risks and benefits, and the possibility of withdrawal at any stage of the experiment, the subjects voluntarily signed the written informed consent approved by the university’s institutional review board.

### 2.2. Supplementation Protocol

Participants self-administered the supplements they were assigned by the researchers for 7 days, after the researcher provided sufficient information on the method and timing of supplement consumption to the participants. The CSB group consumed 20 g/day of creatine monohydrate (Platinum Creatine Powder, MuscleTech, Blasdell, NY, USA) and 0.3 g/kg/day of sodium bicarbonate (Church and Dwight Co., Princeton, NJ, USA). Maltodextrin was assigned to the PLA group (Hammer Nutrition, Ltd., Whitefish, MT, USA) in powder form with similar taste and appearance was consumed at the same dose. Supplements were evenly divided into four separate doses throughout the day (morning, noon, evening, and before bed) to mitigate nausea, belching, diarrhea, and vomiting, which sometimes results from creatine ingestion. Moreover, 5 g of creatine and the specific amount of sodium bicarbonate, calculated based on the individual’s weight divided by 4, were consumed each dose. Supplements were ingested with meals in the morning, afternoon, and evening to increase absorption and storage in the body and with 250 mL of sports drink before bed. The supplementation protocol was based on Barber et al. [19] and Mero et al. [20]. In addition, the subjects were instructed to avoid excessive exercise, physical activity, and caffeine and alcohol intake during the study period.

### 2.3. Soccer-Specific Performance Tests

In this study, soccer-specific performance tests included 10- and 30-m sprints, coordination tests, arrowhead agility tests, and Yo-Yo intermittent recovery level 1 tests. These tests are standard tests currently used to assess soccer players registered with the Korea Football Association. The tests, both pre and post, were conducted over two days. On the first day, 10- and 30-m sprints, coordination test, and arrowhead agility test were conducted. On the following day, Yo-Yo intermittent recovery level 1 test was conducted. Measurements began at 2 p.m. on both days. Measurements began after sufficient fluid intake and warm-up. During each measurement, the participants were instructed to give their maximum effort and were encouraged verbally.

#### 2.3.1. 10- and 30-m Sprints

The 10- and 30-m sprints were factors that could assess the speed and explosive power in the lower limbs of soccer players. At the researcher’s “start” signal, the subjects waiting in a standing position 50 cm behind the start line, sprinted for 10 or 30 m. Timing gates (Brower TC, Brower Timing Systems, Draper, UT, USA) were used to record the sprint times accurately. Timing gates were installed at both the start and finish lines, and an electronic device (Brower TC, Brower Timing Systems, Draper, UT, USA) recorded the time at which the subjects passed the finish line. Each subject’s sprint was measured twice, and the fastest time was used as the final record. All procedures for 10- and 30-m sprints were adopted from Lee et al. [21].

#### 2.3.2. Coordination Test

The coordination test consisted of the subject passing a cone while dribbling a ball. A total of 9 cones were placed over 22 m with a cone at each start and finish line. The first cone was placed 2 m away from the start line, and the cones were placed at intervals of 2 m up to the 4th cone. The remaining 3 cones were placed at intervals of 2 m, starting at a distance of 10 m straight ahead of the 4th cone. Similar to the sprint test, timing gates were placed at both the start and finish lines. The subject waited with the ball in a standing position at the start line and began after the researcher’s signal. The subject dribbled with the ball and passed through a cone located in front in a zigzag manner. After passing all nine cones, the subject returned to the starting line in the same way. If a cone was knocked down, passed, or the turnaround point was not dribbled around properly, 2 s was added to the participant’s time for each error. All coordination test procedures were adopted from Joo et al. [22].

#### 2.3.3. Arrowhead Agility Test

The arrowhead agility test is a highly reliable method for measuring the agility of soccer players [23]. The subject waited in a standing position 50 cm behind the start line and started with the researcher’s signal. The subject sprinted to a cone located 10 m straight ahead, then turned right to run around a cone at a distance of 5 m. After running around the cone, the subject sprinted again to another cone 10 m away from the right cone and sprinted around it back to the start line. Both the right and left sides were measured and performed in the same manner. Each side was measured twice, and the fastest time was used as the final record. The arrowhead agility test procedures were adopted from Lee et al. [21].

#### 2.3.4. Yo-Yo Intermittent Recovery Level 1 Test

The Yo-Yo intermittent recovery level 1 test is useful to measure an athlete’s endurance in intermittent sports in which periods of recovery are combined with high-intensity activities such as soccer [24,25]. It evaluates an individual’s ability to sustain athletic performance across repeated bouts of strenuous exercise [26]. The Yo-Yo intermittent recovery level 1 test was conducted in an outdoor field per Krustrup et al. [25]. Before the test, the participant performed warm-up exercises consisting of light jogging, running, and active stretching for 10 min. The Yo-Yo intermittent recovery test level 1 consisted of a 20 m running zone and a 5 m active recovery zone. Colored cones were placed in each zone to help the athletes concentrate during the test. The subject started with an audio signal from the start line, ran around the cone placed at a 20 m distance, and returned to the finish line before the last audio signal. The subject took a 10-s break in the active recovery zone. The speed increased progressively, and the subject repeated the test following the same method between cones set at 20 m intervals according to the audio signal. If the subject did not pass the finish line within the given time, a warning was given, and the test was terminated after the second warning. The results measured at the end of the test were used as the final records.

### 2.4. Statistical Analysis

SPSS version 18.0 (SPSS, Inc., Chicago, IL, USA) was used for statistical analysis. All data are presented as the mean ± SD. The Shapiro–Wilk, Levene, and Mauchly’s tests were performed for normality, homogeneity, and sphericity of the data. The interactions between performance (pre-and post-supplementation) and group (CSB supplementation and placebo) were analyzed to examine the change in soccer-specific performance after supplementation through analysis of variance (ANOVA) with repeated measures. Significance was set at *p* < 0.05.

## 3. Results

Table 2 shows the changes in 10- and 30-m sprints after CSB supplementation. No differences were observed in the 10 m sprint performance between the CSB and PLA groups (*p* = 0.132, effect size (ES): 0.9). On the other hand, the 30-m sprint time was significantly shorter in the CSB group than in the PLA group (CSB: −3.6% vs. PLA: −0.6%, *p* = 0.018, ES: 2.3). There was no significant change to coordination time after supplementing CSB, nor was there a significant difference in performance between groups (*p* = 0.909, ES: 0.4) (Table 3).

The changes in arrowhead agility (right and left) after CSB supplementation are presented in Table 4. Arrowhead agility was shown to have a statistically significant interaction effect between time and group in both the right (CSB: −7.3% vs. PLA: −0.7%, *p* < 0.001, ES: 2.8) and left (CSB: −5.5% vs. PLA: −1.2%, *p* = 0.001, ES: 2.1). Particularly, in the CSB group, both right and left arrowhead agility time decreased after 7 days. Still, there was no significant difference between pre-and post-intervention in the PLA group. Finally, Yo-Yo intermittent recovery level 1 results are shown in Table 5. These results were not statistically significant (*p* = 0.105, ES: 0.1).

## 4. Discussion

This study examined the effect of 7 days of combined creatine (20 g/day) and sodium bicarbonate (0.3 g/kg/day) supplementation on soccer player’s performance in 10- and 30-m sprints, a coordination, arrowhead agility, and Yo-Yo intermittent recovery level 1 tests. The study results found that the 30-m sprint and arrowhead agility times were significantly improved in the CSB group compared to the PLA group. Sprint and arrowhead agility is an essential component of soccer-specific performance because it is performed at high intensity over a short time and repeatedly implemented during a game [27]. In addition, sprint and arrowhead agility requires power and explosive force and uses energy supplied by anaerobic metabolism [28]. Both creatine and sodium bicarbonate are supplements that can contribute to changes in anaerobic performance.

Creatine increases resting levels of PCr availability to delay PCr depletion during maximal or near maximal exercise and can improve ATP turnover rate [13]. This change can improve anaerobic performance as it helps minimize the decrease in phosphocreatine and the resulting decrease in anaerobic ATP production in high-intensity exercise [10,29]. Previous studies have shown that supplementation with 20 g of creatine per day for 6–7 days can significantly increase the creatine concentration in skeletal muscle and improve anaerobic performance [27,30]. Cox et al. [27] reported that sprint and agility were significantly improved in 12 female soccer players after 6 days of creatine supplementation 4 times a day at 5 g per dose. Ramírez-Campillo et al. [30] also reported that both the repeated sprint and the speed performance in direction change significantly improved in amateur female players after 7 days of creatine supplementation of 20 g per day. Several studies have suggested that the increase in intramuscular creatine phosphate caused by creatine supplementation is a potential cause of improved power in sprints [19,31].

Additionally, Duke and Steele [32] mentioned that PCr depletion could be a potential cause that affects myofilament properties by impairing calcium release through the sarcoplasmic reticulum in skeletal muscles. In relation to this, Sarshin et al. [9] reported that creatine supplementation could increase calcium re-uptake in the sarcoplasmic reticulum to enhance myofibrillar cross-bridge cycling and force development. Moreover, because creatine can remove H+ ions generated during high-intensity exercise owing to increased PCr storage, it can also act as an intracellular buffer [33]. Such action may have contributed to the synergistic effect with sodium bicarbonate in this study. As is well known, sodium bicarbonate supplementation is proposed because of the relationship between exercise-induced acidosis and fatigue [15]. ATP hydrolysis and glycolysis can improve anaerobic performance in soccer because they alleviate metabolic acidosis by removing H+ in cells [7,15].

Meanwhile, it is interesting to note that the Yo-Yo intermittent recovery level 1 test results did not show any difference between the two groups despite the improvements to sprint and arrowhead agility observed in the CSB compared to PLA groups. A positive effect was expected for the Yo-Yo intermittent recovery level 1 test performance, particularly considering the mechanism of sodium bicarbonate in the human body, but no effect was observed. These results contradict Krustrup et al. [17]; they found that sodium bicarbonate supplementation elevated blood alkalosis and peak blood lactate and concomitantly improved the Yo-Yo intermittent recovery level 2 test performance.

The discrepant results may be attributed to the different demands on athleticism required to perform the Yo-Yo level 2 test compared to the level 1 test, as the level 1 test may not have induced intramuscular acidosis sufficiently to observe improvements after supplementing with bicarbonate buffers. In contrast to this study, the Yo-Yo intermittent recovery level 2 test was performed by Krustrup et al. [17]. Unlike the level 1 test, the Yo-Yo intermittent recovery level 2 test demands more anaerobic capacity. Because of this, intracellular acidosis was induced during the test, allowing sodium bicarbonate supplementations to exert their effects. However, since this study did not measure any indicators that can confirm the change in intracellular acidosis, it was not possible to confirm the changes in intracellular acidosis induced by the Yo-Yo intermittent recovery level 1 test.

Rather, the Yo-Yo intermittent recovery level 1 test results in this study introduce the possibility that the improvement of sprint and arrowhead agility was more affected by creatine than by sodium bicarbonate. Sprint and arrowhead agility tests rely on the phosphagen system among the anaerobic metabolic pathways as they are generally performed within 10 s [34,35]. In addition, several studies have shown that sodium bicarbonate supplementation did not affect the changes in repeated sprint performance [36,37]. Recently, Macutkiewicz and Sunderland [38] also reported that sodium bicarbonate did not improve athletes’ sprint or sport-specific skill performance.

This study has several limitations: first, the sample size was small because only one team was selected and tested to avoid confounding influences from different diet and exercise regimes. Second, blood markers, such as lactate or pH, were not measured with creatine or sodium bicarbonate concentrations in the body, which makes any discussion regarding the internal state speculative. In future studies, measuring blood markers may be helpful to be able to determine the internal mechanisms which are affected by supplements. Third, there were few side effects that may appear after CSB supplementation (as mentioned earlier, they include nausea, belching, diarrhea, and vomiting), but such side effects were not closely monitored during the study period with gastrointestinal questionnaire or other methods [39]. Consequently, supplement identification may have appeared in the participants. According to a previous study [40], blinding may be difficult due to side effects that appear after supplementation, which could be a source of bias in sports nutrition. Finally, this study compared the results of only two groups (CSB vs. PLA) without single supplementation groups of creatine or sodium bicarbonate. Although the CSB group showed improved performance compared to the PLA group, it was unknown how much more effective CSB was than creatine or sodium bicarbonate single supplementation groups. These limitations should be addressed in future studies.

## 5. Conclusions

In conclusion, CSB supplementation improved sprint and agility in elite soccer players. However, the results of this study should be interpreted with caution, as it is not yet clear whether the performance improvements is a synergistic effect of the two supplements or a result of either one of them. Thus, it is necessary to add additional control groups so that the effect of CSB supplementation can be confirmed in future studies.

## Figures and Tables

**Table 1:** Physical characteristics of subjects.

Variable	CSB (*n* = 10)	PLA (*n* = 10)	*p*-Value
Age (years)	20.60 ± 0.96	20.80 ± 1.22	0.691
Height (cm)	174.20 ± 3.22	173.70 ± 2.49	0.703
Body weight (kg)	70.68 ± 5.16	69.51 ± 2.38	0.527
BMI (kg/m^2^)	23.18 ± 1.82	22.73 ± 0.98	0.502
Body fat (%)	14.33 ± 2.72	13.62 ± 3.41	0.614

Values are Mean ± SD. CSB: combined creatine and sodium bicarbonate supplementation group; PLA: placebo group; tested by independent *t*-test.

**Table 2:** Change of 10- and 30-m sprint following combined creatine and sodium bicarbonate supplementation.

Variable	Group	Pre	Post	*p*-value	ES
10-m sprint (sec)	CSB (*n* = 10)	1.80 ± 0.06	1.71 ± 0.08	0.132	0.9
	PLA (*n* = 10)	1.83 ± 0.09	1.80 ± 0.11		
30-m sprint (sec)	CSB (*n* = 10)	4.34 ± 0.14	4.18 ± 0.09	0.007 **	2.3
	PLA (*n* = 10)	4.42 ± 0.11	4.39 ± 0.09		

Values are Mean ± SD. ES: effect size, CSB: combined creatine and sodium bicarbonate supplementation group, PLA: placebo group; ** Interaction effect between group and time (*p* < 0.01); tested by analysis of variance (ANOVA) with repeated measures.

**Table 3:** Change of coordination test following combined creatine and sodium bicarbonate supplementation.

Variable	Group	Pre	Post	*p*-Value	ES
Coordination test (sec)	CSB (*n* = 10)	17.37 ± 0.81	17.25 ± 0.65	0.909	0.4
	PLA (*n* = 10)	17.66 ± 0.97	17.56 ± 0.90		

Values are Mean ± SD. ES: effect size, CSB: combined creatine and sodium bicarbonate supplementation group, PLA: placebo group; tested by analysis of variance (ANOVA) with repeated measures.

**Table 4:** Change of arrowhead agility test following combined creatine and sodium bicarbonate supplementation.

Variable	Group	Pre	Post	*p*-Value	ES
Arrowhead agility test (right, sec)	CSB (*n* = 10)	9.14 ± 0.34	8.47 ± 0.26	*p* < 0.001 ***	2.8
	PLA (*n* = 10)	9.26 ± 0.20	9.19 ± 0.26		
Arrowhead agility test (left, sec)	CSB (*n* = 10)	9.25 ± 0.27	8.74 ± 0.32	0.001 ***	2.1
	PLA (*n* = 10)	9.46 ± 0.28	9.34 ± 0.25		

Values are mean ± SD. ES: effect size, CSB: combined creatine and sodium bicarbonate supplementation group, PLA: placebo group; *** interaction effect between group and time (*p* < 0.001); tested by analysis of variance (ANOVA) with repeated measures.

**Table 5:** Change of Yo-Yo intermittent recovery level 1 test following combined creatine and sodium bicarbonate supplementation.

Variable	Group	Pre	Post	*p*-Value	ES
Yo-Yo intermittent recovery level 1 test (m)	CSB (*n* = 10)	2072.00 ± 79.55	2088.00 ± 109.62	0.105	0.1
	PLA (*n* = 10)	2100.00 ± 110.35	2076.00 ± 105.74		

Values are mean ± SD. ES: effect size, CSB: combined creatine and sodium bicarbonate supplementation group, PLA: placebo group; tested by analysis of variance (ANOVA) with repeated measures.

## Data Availability

The data presented in this study are available on request to the authors. Some variables are restricted to preserve the anonymity of study participants.
